# Automatic Quantitative MRI Texture Analysis in Small-for-Gestational-Age Fetuses Discriminates Abnormal Neonatal Neurobehavior

**DOI:** 10.1371/journal.pone.0069595

**Published:** 2013-07-26

**Authors:** Magdalena Sanz-Cortes, Giuseppe A. Ratta, Francesc Figueras, Elisenda Bonet-Carne, Nelly Padilla, Angela Arranz, Nuria Bargallo, Eduard Gratacos

**Affiliations:** 1 Maternal-Fetal Medicine Department, ICGON, Hospital Clınic, Universitat de Barcelona, Barcelona, Spain; 2 Fetal and Perinatal Medicine Research Group, Institut d'Investigacions Biomediques August Pi i Sunyer (IDIBAPS), Barcelona, Spain; 3 Centro de Investigación Biomédica en Red de Enfermedades Raras (CIBERER), Barcelona, Spain; 4 Department of Radiology Hospital Clinic, Centre de Diagnostic per la Imatge, Hospital Clínic, Barcelona, Spain; 5 Image platform IDIBAPS, Barcelona, Spain; King's College London, United Kingdom

## Abstract

**Background:**

We tested the hypothesis whether texture analysis (TA) from MR images could identify patterns associated with an abnormal neurobehavior in small for gestational age (SGA) neonates.

**Methods:**

Ultrasound and MRI were performed on 91 SGA fetuses at 37 weeks of GA. Frontal lobe, basal ganglia, mesencephalon and cerebellum were delineated from fetal MRIs. SGA neonates underwent NBAS test and were classified as abnormal if ≥1 area was <5^th^ centile and as normal if all areas were >5^th^ centile. Textural features associated with neurodevelopment were selected and machine learning was used to model a predictive algorithm.

**Results:**

Of the 91 SGA neonates, 49 were classified as normal and 42 as abnormal. The accuracies to predict an abnormal neurobehavior based on TA were 95.12% for frontal lobe, 95.56% for basal ganglia, 93.18% for mesencephalon and 83.33% for cerebellum.

**Conclusions:**

Fetal brain MRI textural patterns were associated with neonatal neurodevelopment. Brain MRI TA could be a useful tool to predict abnormal neurodevelopment in SGA.

## Introduction

Smallness for gestational age affects 10% of all pregnancies [1]. In clinical practice when an estimated fetal weight is below the tenth centile and Doppler assessment of the umbilical artery is normal, the diagnosis of a small-for-gestational-age (SGA) is reached [2], [3], [4]. Although some fetuses with this diagnosis are constitutionally small, in a substantial proportion of cases, the diagnosis of SGA identifies mild forms of fetal growth restriction due to placental insufficiency that are not expressed by umbilical artery Doppler. Therefore, fetal development occurs in suboptimal conditions, with a deprived delivery of oxygen and nutrients to the fetal brain [5]. Under these conditions brain reorganization may take place, among other changes of the so-called fetal programming [6]. Different authors have shown how despite the fact that most SGA fetuses reach term without signs of deterioration, there is a proportion of them that present an increased risk for an adverse perinatal outcome [7], [8], [9] with an abnormal neonatal neurobehavior [10], [11] and impaired neurodevelopment in early childhood [12]. Considering its prevalence, SGA constitutes a challenge and an opportunity for public health to improve the impact of prenatal conditions in quality of life. However, at present the detection of SGAs at risk of abnormal neurodevelopment is limited since standard clinical examinations fail to identify significant differences. For this purpose, it is crucial to develop new biomarkers based on the characterization of distinctive brain patterns associated with abnormal neurodevelopment. Quantitative imaging based on texture analysis might offer an opportunity for the development of such biomarkers.

Quantitative imaging techniques are based on the application of imaging physics for the development of algorithms improving the information obtained from medical images. These techniques attempt to improve the performance of subjective inspection by extracting quantitative information that may detect non-visible changes and be used in a more objective fashion for prediction, diagnosis and monitoring. Among various approaches, Texture Analysis (TA) is a technique that extracts patterns from images based on the characterization of the microstructural information that may not be assessed visually [13]. It has been widely used in different pathologies [13], [14], [15], [16], [17], being able to classify pathological from healthy tissues in liver [18], breast [17] and tumors [14].We have previously tested a TA software whose efficacy has been shown by different studies on preterm transcranial ultrasound imaging demonstrating a high accuracy in the early identification of preterm white matter damage in subclinical stages [19], on fetal MRIs showing a discrimination based on brain textural features between SGA and AGA fetuses [20] and also when applied on fetal lung ultrasound images, showing a high correlation with gestational age [21]. In a previous study we provided evidence that fetuses with SGA presented statistical differences in their brain MRI textural patterns with respect to controls [20]. In this study we explored whether these patterns showed a correlation with neonatal neurobehavior.

The aim of the study was to test the hypothesis that SGA fetuses show abnormalities in different brain areas reflected by changes in TA, which can be associated to an abnormal neonatal neurobehavior.

## Materials and Methods

### 2.1 Subjects

This study is part of a larger prospective research program on IUGR involving fetal assessment and short and long term postnatal follow-up at the Hospital Clinic (Barcelona, Spain). A prospective cohort of 91 SGA singleton fetuses, defined as an estimated and confirmed birthweight below the 10^th^ centile according to local standards [22] with normal UA pulsatility index (PI) (below the 95^th^ centile ) [23], was included for this study. Exclusion criteria were non-cephalic presentation, the presence of congenital malformations, chromosomal abnormalities, perinatal infections and chronic maternal pathology.

Prenatal and neonatal data were prospectively recorded. The protocol was approved by the institutional ethics committee of the Hospital Clinic of Barcelona and all participants gave written informed consent for exams performed on themselves on the basis of this trial and on their neonates as their legal guardians (Institutional Review Board 2008/4422).

### 2.2 Data acquisition

#### 2.2.1 Ultrasound data

Gestational age was corrected from fetal crown-rump length in the first trimester [24]. Prenatal Doppler ultrasound examinations were performed within one week from MRI scan. Weight estimation, placental and amniotic fluid evaluation were performed using a Siemens Sonoline Antares ultrasound machine equipped with a 6–2 MHz linear-curved-array transducer. Umbilical artery Doppler spectral parameters were obtained automatically from three or more consecutive waveforms with the angle of isonation as close to zero as possible from a free floating cord loop.

#### 2.2.2 Fetal MRI

All cases were scanned at 37 weeks of gestation in a TIM TRIO 3.0 T scanner (Siemens, Germany) without sedation. A body coil with 8 elements was wrapped around the mother's abdomen. Routine fetal imaging took from 15 to 30 min. Fetal neuroimaging consisted on single-shot, fast spin echo T2 weighted sequences (TR 990 ms, TE 137 ms, slice thickness 3.5 mm, FoV 260 mm, voxel size 1.4×1.4×3.5 mm, in plane resolution 192, flip angle 180°, acquisition time 24 seconds) acquired in the three orthogonal planes. If the quality of the images was distorted due to fetal movements, consecutive repetitions were acquired until an acceptable quality image was obtained.

Structural MRI images were reviewed for the presence of anatomical abnormalities by an experienced neuroradiologist, blinded to group membership.

#### 2.2.3 Neurobehavioral performance

Postnatal follow up was offered to all patients. Neonatal Behavioral Assessment Scale (NBAS) test is a standard method for evaluating newborns' capacity to respond to the environment, which reflects brain maturation [25]. It was performed in all 91 patients prospectively at 42±1 weeks by 1 of 2 observers accredited by The Brazelton Institute (Harvard Medical School, Boston, MA) that were blinded to the SGA diagnosis of this group and their perinatal outcomes. This test evaluates 35 items that are rated on a 1 to 9 scale, where 9 is the best performance for some areas and for others this is represented by the central score of 5 [26]. Items are grouped into 6 clusters, including habituation (habituation to light, rattle, bell and tactile stimulation of the foot), motor (general tone, elicited activity, spontaneous activity and motor maturity), social-interactive (responses to visual, animate and inanimate auditory stimuli and alertness), organization of state (irritability, state lability, maximal excitation and reaction time) and regulation of state (self-quieting and hand-to-mouth responses). The social-interactive cluster was subscored for visual and auditory stimuli. In addition, as reported recently by the authors of the NBAS test [27], an aggregation of individual items (alertness, quality of the alert responsiveness and cost of attention) was used to evaluate the capacity of the newborn's attention. Neonates were assessed in the afternoon, between feedings in a small, semidark quiet room with a temperature between 22° and 27°C in the presence of ≥1 parent.

In order to categorize the scores from the studied clusters of the NABS test to determine cases and controls, fifth centile was calculated for each cluster determining in each subject if their performance on that cluster was above or below this centile cutoff.

### 2.3 Classification of the study groups

All 91 SGA neonates that composed our sample were divided into two groups: Cases and controls based on abnormal or normal NBAS test results. SGAs were classified as cases if any of the studied cluster's score (habituation, motor, social-interactive, organization of state, regulation of state and attention) was below the 5^th^ centile, and they were classified as controls if all the scores were above the 5^th^ centile.

### 2.4 Delineation of Regions of Interest (ROIs)

**Figure 1:**
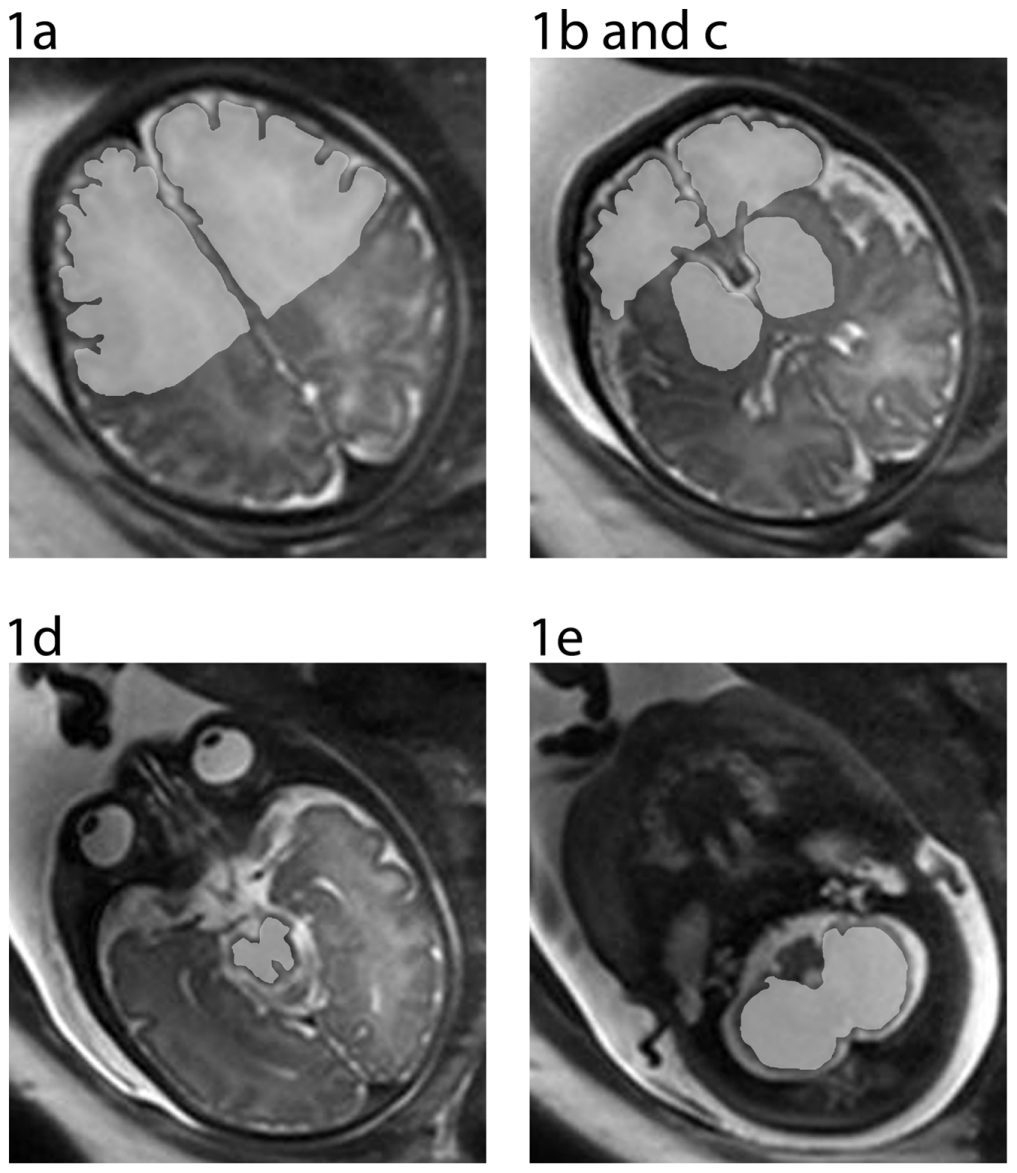
Image selection and ROI delineation: 1a.1b- Right and Left supraventricular frontal lobe; 2a.2b.- Right and Left infraventricular frontal lobe; 3a. 3b- Right and left basal ganglia;4a. 4b.-Right and left Mesencephalon; 5- Cerebellum.

A custom-made Graphical User Interface (GUI) tool on MATLAB R2007b (version 7.5.0.342; MATLAB; the MathWorks Inc., Natick, Massachusetts, USA) was used to manually delineate all nine regions of interest (ROIs). Before delineation, all images were checked for artifacts. If the anatomic area to be delineated showed a suboptimal quality, it was discarded. Delineation was performed by two experienced operators in neuroanatomy blinded to group membership. Right and left supra- and infraventricular frontal lobe, right and left basal ganglia, mesencephalon and cerebellum were selected as clinically relevant ROIs in the studied condition (Figure 1), following the criteria for delineation and image reorientation steps as explained elsewhere [20].

### 2.5 Image analysis

#### 2.5.1 TA and Statistical learning algorithm

The TA method used in the software that was applied is based on wavelet decomposition [28] using Daubechies orthogonal wavelet basis [29]. Wavelets were used to decompose the images in a pyramidal scheme as described by Quellec *et al*. [30] and modified for the use in medical images as previously described [19]. Texture descriptors of an image were described as the concatenation of the marginal distributions of each equalized sub-band image.

This method was applied to the delineated ROIs obtaining a set of 15,300 descriptors per ROI. Based on their anatomic functionality and clinical relevance, descriptors from all 9 delineated ROIs were grouped into 4 main areas:

1. Frontal lobe: left and right infra- and supra ventricular frontal lobe.
2. Basal ganglia: left and right basal ganglia.
3. Mesencephalon: left and right mesencephalon.
4. Cerebellum.

Both infra and supraventricular frontal lobe regions were grouped into one single vector to represent the complexity of the frontal lobe at two different levels. In some cases, one of the ROIs to be merged was not delineated due to an insufficient image quality leading inevitably to a decrease in the number of delineated areas in the frontal lobe. Due to this limitation, we performed our discriminative analysis based on 81 subjects for frontal lobe area, 88 for basal ganglia and mesencephalon and 83 subjects for cerebellum (Table 1).

**Table 1:** Study areas obtained from delineated ROIs.

	Cases (N = 42)	Controls (N = 49)	Total
**Frontal lobe**	38	43	81
**Basal ganglia**	41	47	88
**Mesencephalon**	41	47	88
**Cerebellum**	38	45	83

#### 2.5.2 Selection of descriptors and identification algorithm

Computational models were applied in order to select an appropriate subset of descriptors to identify differences between SGAs with normal or abnormal NBAS test results. To this end, a combination of two artificial intelligence methods were applied: Support Vector Machines (SVM) and Genetic Algorithms (GALs) [31]. As a result, a compact subset of descriptors (between 28 and 77 depending on the area) was automatically selected.

The procedure initially splitted the total sample into two subsets of equal size (subsets “A” and “B”). Firstly, a model was created with subsample “A” and validated with “B”. The accuracy was calculated as the percentage of correctly identified SGAs with normal or abnormal NBAS test results in the validation subset “B”. Subsequently, groups were permuted: a model was created with subsample “B” (using the same subset of descriptors) and validated with “A”, obtaining a second identification percentage. The mean accuracy resulting from the two tests to identify group membership and therefore the possibility of obtaining a normal or abnormal NBAS test was finally measured.

Each model validation result provided a score per subject that was useful for further group comparisons. In order to obtain these scores, the algorithm was designed in a way that the cut-off was assigned to “0”, which is the standard value for SVM. Output values above “0” indicated a high risk for abnormal neurobehavior and below “0” indicated low risk.

### 2.6 Statistical analysis

#### 2.6.1 Demographic and clinical data

Student's t test for independent samples and Pearson's *X* ^2^ or Fisher's exact tests were used to compare quantitative and qualitative data, respectively. Multivariate analysis of covariance was conducted to analyze the results of the NBAS test and the prediction scores for each area between the two groups. For the first analysis a model was carried out for each different set of skills (habituation, motor, organization of state, regulation of state, social interactive and attention) with the study group included as a factor and smoking during pregnancy, gender, Apgar score below 7, days of adaptation from birth to the test and gestational age at the moment of NBAS test as covariates. For the second analysis, a multivariate analysis of covariance was conducted to analyze the results from the prediction scores adjusting for the same covariates.

Results were considered to be significant at p<0.05. All statistical calculations were done using the software package SPSS statistical software, version 17.0(SPSS for Windows, SPSS Inc, Chicago, IL).

## Results

### 3.1 Study groups characteristics

Anthropometric, ultrasound and MRI data were obtained from all patients included in the study. All fetal MR images were considered as normal, not finding signs of intracranial pathology.

As shown in Table 2, when we compared clinical characteristics between SGAs with normal and abnormal NBAS test results, no differences that could explain their different neurobehavioral outcome were found: Both populations were similar in terms of gender, birthweight and biometries. Also, no differences were found concerning signs of perinatal distress or in the rate of breast feeding at discharge or in length of NICU admission (Table 3).

**Table 2:** Maternal characteristics of the population.

	Cases (N = 42)	Controls (N = 49)	*P*
**Maternal age (y)**	31.14±5.9	32.14±5.4	0.41
**BMI (kg/m^2^)**	21.53±3.9	22.0±3.2	0.51
**Primiparity**	83.3	69.4	0.12
**Non-white ethnicity**	26.2	20.4	0.51
**Smoker**	26.2	20.4	0.51
**Superior studies**	43.9	51.1	0.5
**Low -Socio-economic** **status ^a^**	26.5	18.4	0.41
**GA at US (w)**	37.08±1.06	37.2±0.86	0.43
**GA at MRI (w)**	37.25±1.01	37.32±1.01	0.75

Results are expressed as mean ± and standard deviation or percentage determined by Students t-test for independent samples, Pearson's *X* ^2^ or Fisher's exact test as appropriate. Y: years. BMI: Body mass index. GA: Gestational age.US: Ultrasound. MRI: Magnetic Resonance Imaging; w =  weeks. ^a^ Routine occupations, long-term unemployment, or never worked (United Kingdom National Statistics Socio-economic Classification).

**Table 3:** Perinatal outcomes of the population.

	Cases (N = 42)	Controls (N = 49)	P*
GA at delivery (w)	38.79±1.03	38.88±1.26	0.72
Labor induction	76.2	77.1	0.92
Emergency Cesarean section	23.8	30.6	0.46
Cesarean section	26.2	42.9	0.09
Birthweight (g)	2446±289	2422±324	0.70
Birth weight centile	2.45 ±2.74	2.93±2.79	0.41
Male	52.4	65.3	0.21
Head circumference (cm)	32.6±1.14	32.5±2.13	0.74
Length (cm)	46.04±1.79	46.06±2.2	0.95
5 minute Apgar score of <7	2.4	4.1	0.65
Neonatal acidosis ^a^	10	17.4	0.32
Breast feeding during neonatal period	92.7	93.9	0.82
NICU admission	4.8	4.1	0.87
NICU stay length (d)	0.38±1.78	0.12±0.72	0.35

Results are expressed as mean ± and standard deviation or percentage determined by * Student's t-test for independent samples, Pearson's *X* ^2^ or Fisher's exact test as appropriate. GA: Gestational age. NICU: Neonatal Intensive care unit. g =  grams; d = days; w =  weeks. ^a^ Umbilical artery pH <7.15 and base excess >12 mEq/L.

### 3.2 NBAS test results

As shown in Table 4, both populations showed similar adaptation times from birth until the performance of the NBAS test and age at the moment of the test. Concerning the scores of the NBAS test, overall worse results were found in the abnormal NBAS test results group and were more pronounced in the habituation and regulation of state clusters (Table 4).

**Table 4:** Clinical information and results regarding NBAS test.

	Total sample (N = 91)	Cases (N = 42)	Controls (N = 49)	P*
**GA at NBAS test (w)**	42.68±2.82	43.11±2.81	42.73±2.5	0.49
**Adaptation time (d)**	28.44±17.24	30.21±18.06	26.92±16.54	0.36
**Scores from the clusters in the BAS test**				
**Habituation**	6.33±1.34	6.07±1.64	6.57±0.91	0.03
**Social-interactive**	6.17±1.14	5.97±1.23	6.33±1.04	0.32
**Motor**	5.43±0.69	5.33±0.91	5.52±0.43	0.82
**Organization of state**	3.90±0.95	3.73±1.16	4.05±0.71	0.82
**Regulation of state**	4.26±1.38	3.49±1.25	4.93±1.12	< 0.01
**Attention**	6.24±1.58	5.94±1.71	6.5±1.42	0.48

Results are expressed as mean ± and standard deviation. *MANCOVA statistical analysis was used to compare scores in cases vs controls from each area of the NBAS test adjusting for smoking status, gender, Apgar score below 7, gestational age at NBAS test and days of adaptation. GA: Gestational age. w =  weeks; d =  days.

### 3.3 Automatic identification of study group membership based on fetal brain MRI TA

The mean accuracy obtained after the application of the procedure previously explained for each area was 95.56% in basal ganglia area, 95.12% in frontal lobe, 93.18% in mesencephalon and 83.33% in cerebellum.

**Figure 2:**
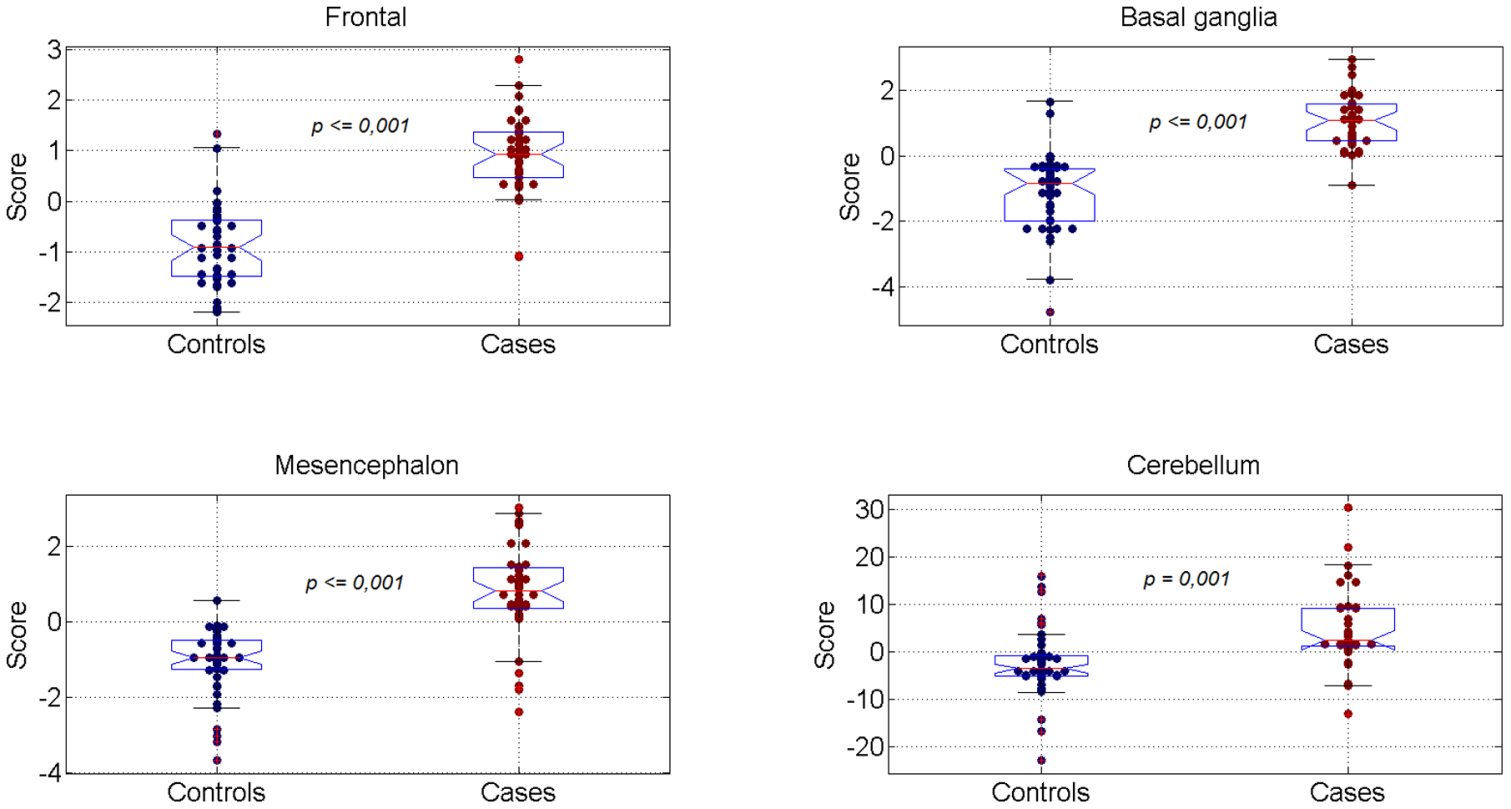
Score distribution of MRI TA-based algorithms for each area under study. Significant differences are present in all areas between cases and controls.*MANCOVA statistical analysis was used to compare scores from each brain area adjusting for smoking status, gender, Apgar score below 7, age at NBAS test and days of adaptation to NBAS test.

There was a significant difference between the scores representing TA for each algorithm output between SGAs with normal and abnormal NBAS test results, in all studied areas. This comparison was adjusted for smoking status, gender, Apgar score below 7, days of adaptation and age at NBAS test. The distribution of the scores obtained with the TA-based algorithms for each area is displayed in Figure 2.

## Discussion

This study provides evidence that fetal brain MRI textural patterns are associated with neonatal neurobehavior and sets the basis for further research on in utero imaging biomarkers based on quantitative assessment of brain microstructure.

The correlation between TA and functional outcome has previously been demonstrated in adults with neurological conditions and apparently normal MRI scans, such as in mild traumatic brain injury or mild cognitive impairment [13], [32]. In these conditions, TA was able to identify differences in relation with the progression of the disease and indicate the most affected areas. To our best knowledge, this is the first time in which brain quantitative imaging in fetuses has been used to establish associations with post-natal neurobehavior. The results are in line with the existence of brain reorganization in IUGR. Different lines of evidence have shown that fetuses and infants affected with early and severe IUGR have significant differences in brain metabolism, sulcation, composition, and microstructure [33], [34], [35], [36]. Furthermore, correlations between these brain disturbances and neurological performance have been reported [33], [35].The majority of earlier studies were conducted in early-onset IUGR. However recent evidence supports that late-onset IUGR have changes in the same direction, including differences in brain metabolism and microstructure [37] and signs of increased axonal loss at 5 years of age [38]. Along the same lines, in a previous study we reported differences in textural patterns on fetal brain MRI between term SGA and AGA fetuses [20]. In the present study we provide evidence that these patterns are correlated with post-natal neurobehavior.

From a pathophysiological point of view, textural patterns could reflect brain microstructural alterations in late-onset IUGR fetuses. Brain reorganization is thought to underlie developmental deficits of SGA infants, which show cognitive disadvantages from the neonatal period until adolescence [10] [39], [40]. It is increasingly accepted that subtle changes in brain morphology may be present years before the clinical onset of neuropsychiatric and neurodegenerative diseases [41], [42].These changes could be identified by quantitative imaging in order to define “early endophenotypes” as markers of future functional outcome [33]. Therefore, results obtained from this study encourage further research aiming at the identification of such “imaging endophenotypes” in IUGR, and possibly other neurocognitive disorders of fetal and perinatal origin.

In this study we chose several brain areas that might potentially be involved in brain reorganization affecting neurodevelopment. Attention skills are generally attributed to the frontal lobe, due to its importance for cognitive tasks and the results of MRI studies of attention deficit and hyperactivity disorder [43]. On the other hand, potential cerebellar microstructure alterations could be preferentially involved in lower scores of the motor cluster, including motor learning, memory and cognition and in behavior [44]. However, brain neurostructure and organization undergoes substantial changes during the two first years of age, and in general extrapolation of observations from older children or adults to fetal and perinatal life is not feasible. In this study we did not find definite correlations between specific areas and behavioral domains. Actually, basal ganglia, frontal lobe and mesencephalon obtained similarly high accuracies in predicting their neurobehavioral outcome. As mentioned, this was somewhat expected. The contribution from each brain area to the NBAS test is unknown, probably existing direct or indirect influences from all areas in various NBAS clusters at this primitive stage of neurodevelopment.

From a clinical perspective, the study provides further evidence to support the existence of changes in brain development, which could be used for diagnosis of true forms of fetal growth restriction in utero. Identifying at-risk patients lays the basis for timely interventions in utero to decrease the rate of adverse perinatal results [8] and for selection of newborns for targeted interventions. Evidence from randomized trials indicates how preterm-born IUGR neonates that received the Newborn Individualized Developmental Care and Assessment program (NIDCAP) showed better neurobehavior, electrophysiology and brain structure than those receiving standard care [45]. Other interventions with demonstrated impact include breast feeding, with a positive effect on brain white matter growth [46] and a worse adherence in IUGR newborns due to a poorer regulation and organization of state during the neonatal period [47]. The potential clinical value of TA in the identification of risk requires a great deal of further research. At this point, most quantitative imaging-based methods are still far from clinical applications. Specifically, TA-based applications require developing robust algorithms based on large databases, software user interface platforms and feasibility studies demonstrating its value in clinical practice, and it is likely to be years before these studies are completed.

One strength of this study is that it evaluates brain MRI TA from a homogeneous cohort of term SGA fetuses selected in utero and prospectively followed up until the neonatal period. The correlation with neurobehavioral scores weeks after birth supports the importance of prenatal factors as a strong independent contributor to neurodevelopment, irrespective of postnatal events. It was remarkable that there was not any a priori potential bias on NBAS examiners since SGA newborns with normal and abnormal NBAS were homogeneous with respect of weight and length. In addition, study groups were similar in terms of perinatal outcomes and other potential confounding factors, such as days of adaptation, breast feeding or educational level from the mother.

However, we grant some limitations and technical considerations in this study. We acknowledge that this study based its functional outcome on neonatal neurobehavioral scores and not in long term cognitive evaluation. However, increasing evidence supports a neurobiological basis for infant or neonatal behavior [48], linking neonatal neurobehavioral skills with later neurocognitive development [25], [36], [47], [49], [50] and showing how scores on neurobehavioral tests predict IQ at 6 years of age [25].

Concerning the statistical learning algorithm that was developed for this study, it should be stated that the limited sample size prevented the use of an external validation by an independent group. However, the two-fold cross validation used can minimize the impact of this limitation. We acknowledge that these results are preliminary and require confirmation in larger sample sizes allowing external validation.

In summary, this study provides evidence that fetal brain quantitative imaging based on MRI TA has a potential in predicting an abnormal neurobehavioral outcome. This study supports further research on quantitative imaging techniques to develop imaging biomarkers of abnormal neurodevelopment in late-onset IUGR fetuses.
